# Optimization of Enzyme-Assisted Extraction of Bioactive Compounds from Sea Buckthorn (*Hippophae rhamnoides* L.) Leaves: Evaluation of Mixed-Culture Fermentation

**DOI:** 10.3390/microorganisms11092180

**Published:** 2023-08-29

**Authors:** Viktorija Puzeryte, Paulina Martusevice, Sérgio Sousa, Aiste Balciunaitiene, Jonas Viskelis, Ana Maria Gomes, Pranas Viskelis, Laima Cesoniene, Dalia Urbonaviciene

**Affiliations:** 1 Institute of Horticulture, Lithuanian Research Centre for Agriculture and Forestry, 54333 Kaunas, Lithuania; viktorija.puzeryte@lammc.lt (V.P.); paulina.martusevice@lammc.lt (P.M.); aiste.balciunaitiene@lammc.lt (A.B.); jonas.viskelis@lammc.lt (J.V.); pranas.viskelis@lammc.lt (P.V.); 2 Botanical Garden, Vytautas Magnus University, Z.E. Zilibero 6, 46324 Kaunas, Lithuania; laima.cesoniene@vdu.lt; 3 Universidade Católica Portuguesa, CBQF-Centro de Biotecnologia e Química Fina-Laboratório Associado, Escola Superior de Biotecnologia, Rua Diogo Botelho 1327, 4169-005 Porto, Portugal; sdsousa@ucp.pt (S.S.); amgomes@ucp.pt (A.M.G.); 4 Research Institute of Natural and Technological Sciences, Vytautas Magnus University, 40444 Kaunas, Lithuania

**Keywords:** sea buckthorn leaves, *Hippophae rhamnoides* L., fermentation, enzyme-assisted extraction, optimization, central composite design

## Abstract

*Hippophae rhamnoides* L. leaves possess a remarkable amount of polyphenols that could serve as a natural remedy in various applications. In comparison, numerous techniques, such as conventional and high-pressure techniques, are available for extracting the bioactive fractions from sea buckthorn leaves (SBL). However, enzyme-assisted extraction (EAE) of SBL has not been comprehensively studied. The aim of this study was to optimize critical EAE parameters of SBL using the cellulolytic enzyme complex, Viscozyme L, to obtain a high-yield extract with a high concentration of bioactive compounds. In order to determine the optimal conditions for EAE, the study employed a central composite design and response surface methodology to analyze the effects of four independent factors (pH, temperature, extraction time, and enzyme concentration) on two different responses. Our findings indicated that under optimal conditions (3:15 h extraction, temperature 45 °C, pH 4.9, and 1% Viscozyme L *v*/*w* of leaves DW), EAE yielded 28.90 g/100 g DW of the water-soluble fraction. Furthermore, the EAE-optimized liquid extract was continuously fermented using an ancient fermentation starter, Tibetan kefir grains, which possess lactic acid bacteria (LAB) and have significant potential for use in biopreservation. Interestingly, the results indicated various potential prebiotic characteristics of LAB. Additionally, alterations in the cell wall morphology of the SBL residue after EAE were examined using scanning electron microscopy (SEM). This study significantly optimized EAE parameters for sea buckthorn leaves, providing a promising natural source of bioactive compounds for various applications, such as nutraceuticals, functional foods, and high-value products.

## 1. Introduction

Sea buckthorn (*Hippophae rhamnoides* L.) belongs to the family Elaeagnaceae [1]. It is a hardy shrub that grows in harsh environments, such as high altitudes and sandy soil, and it is widespread in various locations in Europe and Asia [1,2,3]. It is an essential plant used for centuries by many cultures as a traditional medicine and food source [3,4]. Sea buckthorn is well-known for its fruits, which are rich in bioactive compounds such as vitamins, phenolic acids, flavonoids, carotenoids, and tocopherols [5].

Recently, increased focus has been placed on exploring the bioactive compounds, antioxidant benefits, and potential applications of sea buckthorn leaves (SBL) as a by-product of fruit harvesting and processing [6,7]. It is important to mention that the leaves of the sea buckthorn plant contain a substantial quantity of bioactive compounds, including phenolic acids, flavonoids, carotenoids, and vitamin C [7,8,9]. According to scientific research, these compounds have been found to possess remarkable antioxidant, antiviral, antitumor, anti-inflammatory, and antimicrobial properties [4,10,11]. SBL is a promising potential ingredient due to its nutritional and medicinal components, which offer numerous health benefits to humans. These compounds make SBL a potential ingredient for various applications [11,12].

There is enormous potential in effectively utilizing food waste and by-products to obtain functional ingredients [13]. The efficient extraction of these ingredients through methods such as enzyme-assisted extraction (EAE) presents promising opportunities [14]. Briefly, enzymatic technology utilizes enzymes as catalysts to break down plant cell wall components by binding the desired substrate to the enzyme’s active site, which increases accessibility to carbohydrates, proteins, and phenolic compounds and their nutritional value [15,16]. For instance, EAE has the ability to extract pectin from waste and by-products by enhancing the permeability of the plant cell wall [13,17,18]. By implementing this technique, it is possible to increase the yields of extracts within a shorter extraction time and reduce solvent use while simultaneously enhancing the concentration of bioactive compounds such as various phenolic compounds, including flavonoids and anthocyanidins [19]. This approach also minimizes production costs and time requirements while ensuring that environmental sustainability is prioritized throughout the process [15].

However, in order to obtain extracts that have increased yields and improved properties, it is necessary to have a comprehensive understanding of the hydrolytic properties of the enzymes used and their physicochemical interactions with the raw materials [15]. Therefore, it is essential to optimize critical parameters of the EAE process, such as pH, temperature, and enzyme concentration, for every plant material and its morphological part being extracted. Due to different ratios of specific glycoside, sulfur, and other bonds in plant cell walls, the chosen enzymes significantly impact the release of bioactive substances.

As mentioned, hydrolytic enzymes cleave lignocellulose material, releasing trapped molecules in the cell wall such as reducing sugars and oligosaccharides of various lengths [20]. Current articles describe increasing the prebiotic and probiotic potential of foods and beverages using different plant materials and starter cultures, respectively. Spontaneous or mixed-culture fermentation is gaining popularity due to its symbiotic and complex beneficial outcomes that contribute to health [21,22,23,24]. The best-known mixed-culture starters include *Medusomyces gisevii* pellicle, kefir grains, and birch sap, which implement bacterial and yeast symbiosis [25]. Although scientific investigations of mixed-culture starters are limited, the highest LAB content was identified for kefir grains, especially Tibetan kefir grains (TKG) [26,27,28,29,30].

TKG is a traditional symbiotic culture of bacteria and yeast [31]. It has been used for centuries to produce kefir [26,32] and is currently introduced in various fermented plant-based beverages [33]. An investigation of grain-based beverages fermented with Tibetan kefir grains conducted by Streimikyte et al. in 2022 identified LAB commonly used in yogurt production for growth and possible prebiotic potential. The results showed that the LAB content was higher than the Codex Alimentarius recommendation of 10^7^ CFU mL^−1^, which describes a dairy yogurt standard. The same paper identified the antimicrobial properties of plant-based fermented beverages, which had variable pathogenic strains. These variations suggest a possible differentiation in the metabolomics of fermented products and their pathways in pursuing biopreservation characteristics [20,34,35,36].

**Figure 1:**
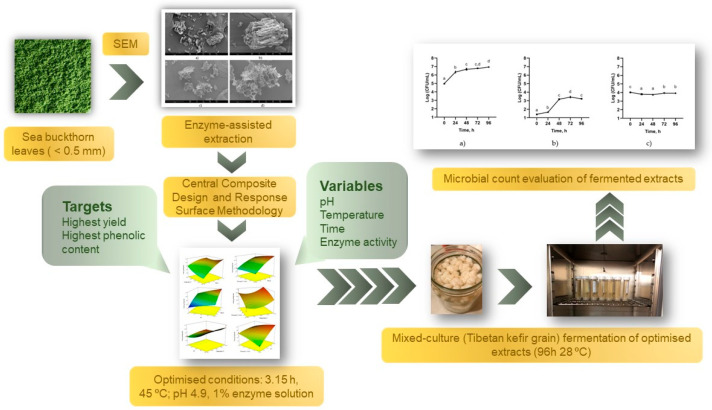
Overview of the experimental design.

This study aimed to optimize the bioactive compound content and yield of SBL extract using EAE with the cellulolytic enzyme mixture, Viscozyme L. Viscozyme L can effectively disintegrate plant matrices, resulting in a higher extraction yield and increased levels of phenolic compounds. Additionally, the study aimed to determine the optimal EAE conditions using a central composite design (CCD) and response surface methodology (RSM). The study aimed to obtain a high-concentration and high-yield extract of bioactive compounds that could be used in various applications, such as nutraceuticals, functional foods, and high-value products. Additionally, the study investigated alterations in the cell wall morphology of the residue before and after EAE, as well as the feasibility of fermenting the optimized extract using Tibetan kefir grains for potential use in biopreservation and its prebiotic potential, which is described in the graphical overview shown in Figure 1.

## 2. Materials and Methods

### 2.1. Plant Material

Sea buckthorn plants were grown in the experimental fields of the Institute of Horticulture, Lithuanian Research Centre of Agriculture and Forestry (55°08′ N, 23°80′ E). Fresh SBL was harvested in 2021. The collected leaves were immediately frozen at −35 °C and then freeze-dried. The samples were freeze-dried using a Zirbus lyophilizer (Zirbus Technology GmbH, Bad Grund, Germany) at 0.01 mbar pressure and a condenser temperature of −85 °C. The freeze-dried samples were ground to a powder using a Retsch 200 knife mill (Haan, Germany) and stored in a sealed container until analysis.

### 2.2. Chemicals, Reagents, and Enzyme Products

Folin–Ciocalteu phenol reagent, gallic acid (3,4,5-trihydroxybenzoic acid, 99%) and Na_2_CO_3_ were purchased from Sigma-Aldrich (Steinheim, Germany).

Viscozyme L is a cellulolytic enzyme complex derived from *Aspergillus aculeatus*. It was purchased from Sigma-Aldrich (Steinheim, Germany). Furthermore, the manufacturer states that the enzyme mixture contains a wide range of arabanase, cellulase, β-glucanase, hemicellulase, and xylanase. The product is declared to have ≥100 FBGU/g.

### 2.3. Central Composite Design (CCD) and Response Surface Methodology (RSM)

Central composite design (CCD) and response surface methodology (RSM) were used to obtain the optimal enzyme-assisted extraction (EAE) conditions for yield and total phenolic compounds of SBL. For data analysis and the established data, Design-Expert 7.0.0 software (Stat-ease, Inc., Minneapolis, MN, USA) was used to determine the models and analyze the results. Four independent variables, time (1–4 h), temperature (40–60 °C), pH (4.5–6), and Viscozyme L enzyme concentration (0.5–3%/g dry leaves), were examined at three points (axial, center, factorial) with different levels (−α, −1, +1, +α), as shown in Table 1. In addition, the desirability function was implemented to describe and obtain an optimized multiple-method response. All variables were chosen within the range, but yield and total phenolic content (TPC) were selected as maximized goals. The model’s suitability was assessed by analyzing the determination coefficient, adjusted R-squared value, and anticipated R-squared value.

### 2.4. Enzyme-Assisted Extraction (EAE)

EAE was carried out as described by Streimikyte et al. [19], with minor adjustments. SBL samples were extracted using hot water EAE at a 1:20 (*w*/*v*) ratio and mixed to obtain a homogeneous suspension. The suspension pH (3.0–5.0) was adjusted using 6 M HCl and 0.5 M NaOH, and enzyme Viscozyme L (0.5–3% *v*/*w*) was added. EAE was carried out at 40–60 °C in an incubator. After extraction, the enzyme was deactivated by heating the hydrolyzed material at 95 °C for 10 min. Then, separation of the suspension into liquid and solid fractions was accomplished using a filter (200 mesh). Samples were freeze-dried and stored at room temperature. Afterward, the obtained extracts were re-extracted for further analysis. All experiments were conducted in triplicate.

### 2.5. Spontaneous Fermentation Using Tibetan Kefir Grains

Following EAE, the liquid phase was used for further fermentation before freezing, as previously described by Streimikyte [28]. Specifically, 10% Tibetan Kefir grains was added to the liquid samples. The fermentation process was carried out in an incubator for four days (96 h) while maintaining a consistent temperature of 28 °C. Upon completion, the fermented samples were filtered and subsequently frozen at a temperature of −20 °C for further analysis.

### 2.6. Measurement of pH

The pH was obtained using a model MW102 pH meter with an MA 920 electrode (Milwaukee, Sat Baciu, Romania).

### 2.7. Determination of Total Phenolic Content

The total polyphenol content (TPC) in the hydrolyzed extracts after EAE was determined according to the Folin–Ciocalteu method [37], using gallic acid (GA) as the standard, as described by Bobinaite et al. [38]. The reagent was prepared by diluting a stock solution with ultra-pure distilled water (1/10, *v*/*v*). Samples (1.0 mL, three replicates) were introduced into test cuvettes, followed by 5.0 mL Folin–Ciocalteu’s phenol reagent and 4.0 mL Na_2_CO_3_ (7.5%). The system was then placed at ambient temperature for 1 h. The absorbance of all samples was measured at 765 nm using a Cintra 202 spectrophotometer (GBC Scientific Equipment, Knox, Australia). The total phenolic content was determined from the calibration curve and expressed in mg gallic acid equivalents in 100 mL extract.

### 2.8. Scanning Electron Microscopy (SEM)

The ultrastructure and morphological alterations in the SBL residue before and after EAE were analyzed using scanning electron microscopy (SEM). Freeze-dried SBL samples before and after EAE were fitted on double-sided adhesive carbon tape (NEM tape; Nisshin, Tokyo, Japan) mounted on SEM pins, sputter coated with gold/palladium (Polaron, Germany), and visualized using a Phenom^TM^ XL G2 SEM (Thermo Fisher Scientific, Eindhoven, The Netherlands). Analyses were performed at an accelerating voltage of 5 kV using a secondary electron detector (SED).

### 2.9. Microbial Evaluation of Fermented Samples

To assess the growth of *Lactobacillus delbrueckii* subsp. *bulgaricus* and *Streptococcus thermophilus* in spontaneously fermented samples with TKG, microbial evaluation was performed using agar diffusion [28]. Wells 6 mm in diameter were punched in the agar and filled with fermented extracts. The agar plates were incubated at 37 °C for 24 h, and the colonies were counted and expressed in Log (CFU/mL). Viable mesophilic lactic acid bacterial counts were also determined through serial dilution and plating.

### 2.10. Statistical Analysis

All analyses were performed in triplicate. MS Excel 2020 (Redmond, WA, USA) was used to calculate mean values and standard deviations. One-way analysis of variance (ANOVA) was performed on the response surface results using Design-Expert 7.0.0 software (Stat-Ease Inc., Minneapolis, MN, USA). ANOVA followed by Turkey’s HSD test was employed for statistical analysis at a probability level of *p* < 0.05.

## 3. Results and Discussion

### 3.1. Optimization of Enzyme-Assisted Extraction (EAE)

This study sought to assess and improve EAE as a productive method of valuing SBL. SBL biomass is rich in bioactive compounds but is usually regarded as waste, although obtained EAE extracts could be interesting in various sectors. The key benefits of EAE are cost-effectiveness, environmental friendliness, and the ability to release phenolics from their insoluble bonds as more bioactive components [14,19,39]. Multiple authors have suggested RSM and CCD to assess and optimize enzymatic hydrolysis factors such as temperature, time, enzyme concentration, and pH [19,40,41]. In this research, the effects of four independent variables, including time (min.), enzyme concentration (% *v*/*w*), temperature (C), and pH, on EAE extract yield (g/100 g DW) and TPC (mg GAE/100 mL extract) were determined by applying RSM and CCD. The experimental conditions investigating the independent variables were chosen based on an earlier publication [19].

The developed models were evaluated for adequacy using analysis of variance (ANOVA). The results indicated that all of the proposed models were significant (*p* < 0.0001), and there was no significant “lack of fit” relative to the pure error (*p*-value of 0.1045). The determination coefficient R-squared value of 0.9251 indicated a good fit of the model to the experimental data. In addition, the predicted R-squared value of 0.7657 was reasonably consistent with the adjusted R-squared value of 0.8502. This level of precision suggested that the models could be used to effectively navigate the design space. Additionally, the reproducibility of the models could be considered reasonable, as evidenced by the coefficient of variation (CV) value of 1.93%. The results suggested that the developed models were suitable for further analysis and application. The polynomial regression model equations provided the empirical relationships between the dependent and independent variables:

Yield = +25.22 + 0.86 × A + 0.30 × B + 0.29 × C − 0.49 × D − 0.54 × A × B − 0.22 × A × C − 0.13 × A × D − 0.019 × B × C + 0.049 × B × D + 0.53 × C × D − 0.38× A^2^ + 0.41 × B^2^ − 0.14 × C^2^ − 0.13 × D^2^

TPC = +254.10 − 2.94 × A − 14.49 × B + 5.09 × C − 17.87 × D − 24.10 × A × B + 5.75 × A × C − 27.41 × A × D − 2.07 × B × C − 24.17 × B × D + 7.28 × A^2^ − 2.15 × B^2^ + 1.26 × C^2^ + 0.91 × D^2^

where A = time (1–4 h), B = temperature (40–60 °C), C = enzyme concentration (0.5–3% *v*/*w*), and D = pH (4.5–6).

The effects of four independent variables on extract yield and total phenolic content are presented in Table 2.

**Figure 2:**
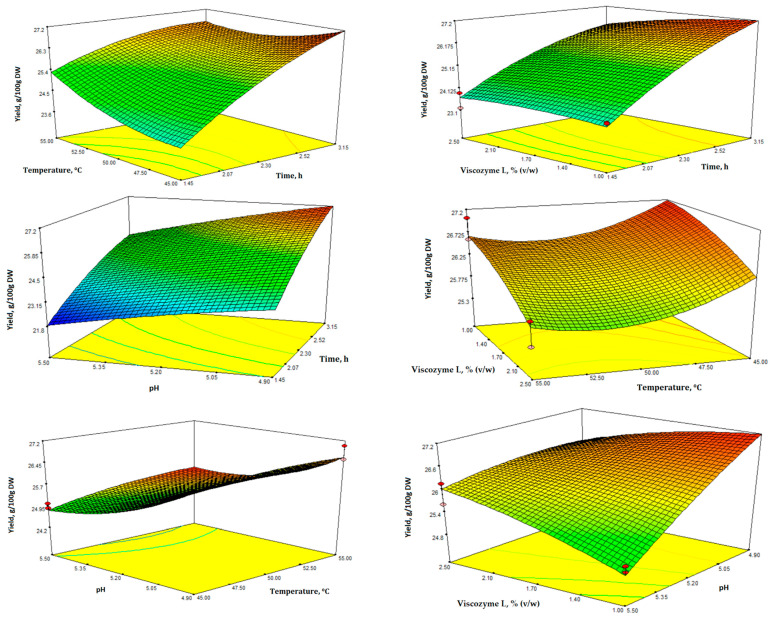
Response surface 3D plots for extraction yield (g/100 g DW) showing the effects of the four independent variables (temperature, time, enzyme concentration, and pH).

**Figure 3:**
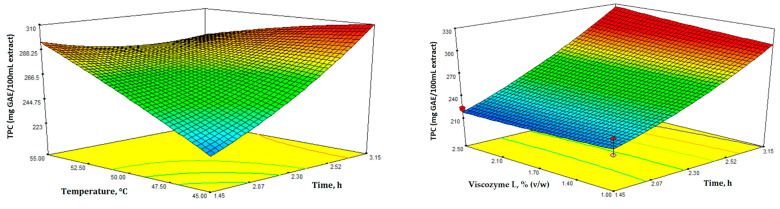

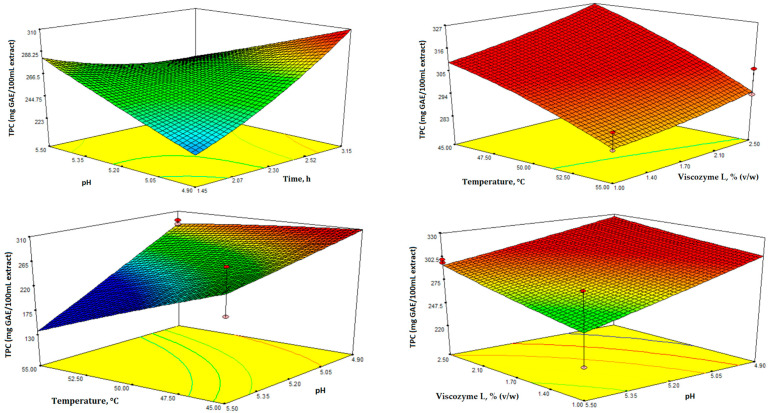
Response surface 3D plots for total phenolic content (mg GAE/100 mL extract) showing the effects of the four independent variables (temperature, time, enzyme concentration, and pH).

In Figure 2, the 3D plots display the impact of the four variables and the interactions between various tested factors. These plots provide insight into the significance of the four factors on EAE extract yield and TPC (Figure 2 and Figure 3).

The interaction contour plots revealed a notable impact of all independent variables on the yield of the water-soluble fraction after EAE of SBL. As expected, there was a clear and positive relationship between yield and longer extraction time, as well as lower enzyme concentration and temperature (Figure 2). According to the results, the yield of the water-soluble fraction varied significantly, ranging from 21.87 to 27.43 g/100 g of leaves DW (Table 2).

In Figure 3, the 3D response surface plots depict TPC as a function of the different independent variables. TPC was determined using the Folin-Ciocalteu assay and measured in mg of GAE/100 mL extract. Additionally, the TPC values ranged from 209.10 to 304.80 mg GAE/100 mL extract (Table 2).

The ANOVA results indicated that the suggested models for yield and TPC were suitable for predicting the correlation between the extraction process parameters and the different responses within the chosen experimental domain. EAE was meticulously fine-tuned using numerical optimization and the desirability function within the selected range of variables by thoroughly analyzing all responses. Design-Expert software was utilized to consolidate the various responses and factors into a single desirability function, which enabled numerical optimization to identify the optimal point that maximized overall desirability while adhering to the specified constraints. The four experimental independent variables, enzyme concentration, pH, temperature, and time, were set as “in range” for this study. In order to achieve optimal enzyme activity and specificity, it is imperative to consider several critical factors, including temperature, pH, and the appropriate concentrations of enzymes and substrates [42,43]. Based on the specified criteria, applying the desirability function enabled the simultaneous optimization of all responses. The recommended optimal extraction conditions were a temperature of 45 °C, pH of 4.9, extraction time of 3:15 h, with an enzyme concentration of 1% *v*/*w* of leaves. Under these conditions, the overall desirability was 0.978, with the highest water-soluble fraction yield (27.19 mg/100 g DW) and total phenolic content (309.54 mg GAE/100 mL extract).

### 3.2. Scanning Electron Microscopy Analysis of Plant Material before and after EAE

**Figure 4:**
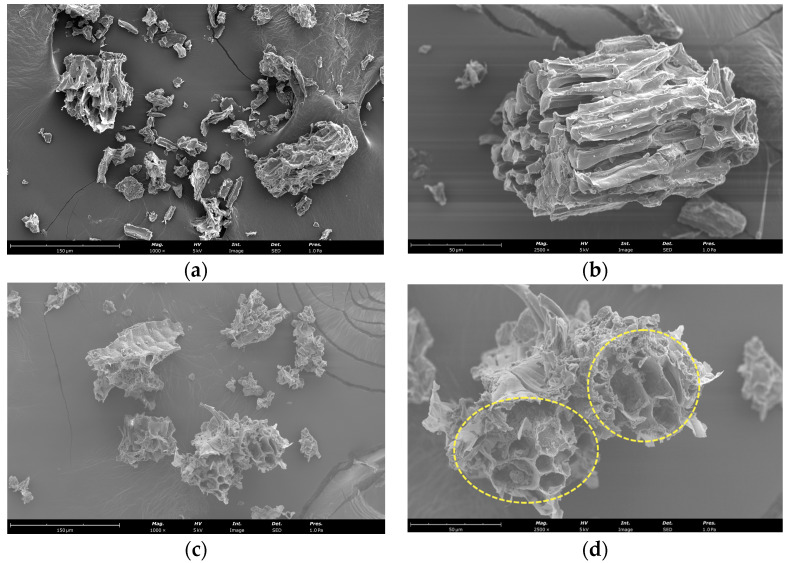
SEM micrographs of SBL before (**a**,**b**) and after EAE (**c**,**d**). The images are at 1000× and 2500× magnification, and scale bars represent 150 and 50 µm (**a**,**c** and **b**,**d**, respectively). Dashed yellow circles highlight morphological alterations.

Scanning electron microscopy was employed to assess the impact of EAE on cell wall degradation. As depicted in Figure 4, the microstructure of the plant material exhibited distinguishable disparities following treatment with Viscozyme L. The control sample (Figure 4a,b) portrayed a smooth surface without any ruptures or significant disruption to the microstructure. After applying Viscozyme L, a significant amount of tissue fragments was observed (Figure 4c,d), which were covered with numerous small particles. Regarding the cell surface, partial exfoliation and altered morphology were detected. Following fermentation, the surface displayed a rough, uneven texture and was prone to destruction. Figure 4d shows the opening and formation of single particles, supporting the occurrence of fermentation and the associated morphological alterations.

The complexity of plant cell walls plays a critical role in providing structural support and protection against the release of intracellular components [44]. Bioactive compounds can exist in both bound and free forms, and enzymatic breakdown of the cell wall can significantly increase the yield of these compounds and enhance the antioxidant capacity [45,46,47], as evidenced by morphological changes (Figure 4). Previous studies of by-products have demonstrated noticeable morphological alterations in cell walls following the enzymatic hydrolysis process [48,49].

### 3.3. Microbial Count of Sea Buckthorn Leaves Extract Fermented with Tibetan Kefir Grains

Improving the nutritional value and sensory properties of fermented products is crucial, and this can be achieved by producing metabolites such as organic acids (especially lactic acid). Additionally, microorganisms (particularly LAB) play a vital role in extending the shelf-lives of products by reducing the pH value through their metabolic activity [50,51]. The quality of a product is greatly influenced by the viability of microorganisms, which significantly impacts gut colonization and microbiota development [52]. The widely recognized yogurt starters, *Streptococcus thermophilus* and *Lactobacillus delbrueckii* subsp. *bulgaricus*, are used in the fermentation of plant-based products [28,53,54]. In different studies, these strains have been found to alleviate the symptoms associated with lactose intolerance [55], which is related to the provision of β-galactosidase (lactase), a digestive enzyme that aids in breaking down lactose [56,57]. This property confers strain-dependent probiotic potential. These microbial strains represent a fraction of the LAB content.

**Figure 5:**
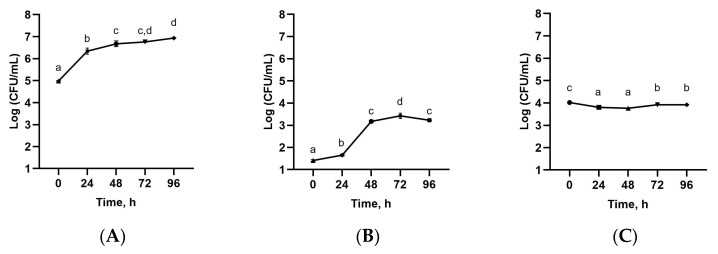
Evolution of viable cell numbers of LAB in enzyme-assisted extracts fermented with TKG; (**A**) mesophilic lactic acid bacteria; (**B**) *Streptococcus thermophilus*; (**C**) *Lactobacillus delbrueckii* subsp. *bulgaricus*. Different letters indicate significant differences (one-way ANOVA and Tukey’s HSD test, *p* < 0.05).

Following EAE, the hydrophilic extracts were gathered and prepared for further fermentation with TKG. Accordingly, the fermentation was studied over time for five days (0, 24, 48, 72, 98 h), during which time samples were filtered, the pH was measured (Table 3), and the viable cell numbers of mesophilic lactic acid bacteria (*Streptococcus thermophilus* and *Lactobacillus delbrueckii* subsp. *bulgaricus*) were counted (Figure 5). During the fermentation kinetics, the pH values decreased at 24 and 48 h with pH values of 4.68 ± 0.021 and 4.71 ± 0.041, respectively, and then remained stable (Table 3). The pH level decreased because microorganisms produced organic acids that lowered the pH to less than 4.0 [58,59]. The pH values were also in agreement with the viable cell numbers of mesophilic lactic acid bacteria (*Streptococcus thermophilus* and *Lactobacillus delbrueckii* subsp. *bulgaricus*) during fermentation (Figure 5).

The viability of mesophilic lactic acid bacteria (*Streptococcus thermophilus* and *Lactobacillus delbrueckii* subsp. *bulgaricus*) in the fermented extracts is presented in Figure 5. The highest viable cell numbers for total mesophilic lactic acid bacteria, as well as for *Streptococcus thermophilus*, were obtained after four days of fermentation. On the other hand, the growth *of Lactobacillus delbrueckii* subsp. *bulgaricus* was not statistically significant. It should be pointed out that the total mesophilic lactic acid bacteria counts remained close to the recommended levels for traditional kefir, i.e., >10^7^ CFU/mL [60], which was in agreement with previous reports of plant-based fermentation with TKG [50,61]. The reported bacterial growth may be related to the prebiotic effect of polysaccharides and bioactive compounds in SBL extract [62,63].

## 4. Conclusions

This research aimed to obtain a high-concentration and high-yield extract of bioactive compounds via EAE of SBL, which could be used in various applications, such as nutraceuticals, functional foods, and high-value products. The optimized conditions identified the highest desirability after 3:15 h of extraction, a temperature of 45 °C, pH of 4.9, and 1% Viscozyme L (*v*/*w*). Moreover, SEM images identified successful cleavage and hydrolysis by hydrolytic enzymes. Although further studies are needed, the results were promising for total mesophilic LAB and *Streptococcus thermophilus*, identifying SBL extract as a potential prebiotic for selected probiotic strains.

## Figures and Tables

**Table 1:** Five levels of the four variables of the extraction process.

Variable	Units	Symbol	Coded Levels				
			−α	−1	0	+1	+α
Time	h	A	1:00	1:45	2:30	3:15	4:00
Temperature	°C	B	40	45	50	55	60
Viscozyme L	%	C	0.5	1.0	2.0	2.5	3.0
pH	-	D	4.5	4.9	5.3	5.6	6.0

**Table 2:** Comprehensive central composite design matrix for the enzyme-assisted extraction of sea buckthorn leaves and results on yield and total phenolic content.

	Time	Temperature	Viscozyme L	pH	Yield	TPC
Run	min	°C	%		g/100 g DW	mg GAE/100 mL Extract
1	2:30	50	3.0	5.3	25.80	258.30
2	1:45	55	2.5	5.6	25.75	266.67
3	2:30	50	2.0	5.3	25.86	257.30
4	2:30	50	2.0	5.3	25.72	261.90
5	3:15	45	2.5	5.6	26.17	296.00
6	1:45	45	1.0	4.9	23.86	237.23
7	1:45	45	2.5	4.9	23.19	224.30
8	2:30	50	2.0	4.5	25.66	286.05
9	2:30	50	2.0	6.0	23.71	214.55
10	2:30	50	2.0	5.3	25.27	258.13
11	1:45	55	2.5	5.6	25.29	264.43
12	2:30	40	2.0	5.3	26.23	267.05
13	3:15	45	2.5	5.6	25.61	299.50
14	3:15	55	2.5	4.9	25.79	304.80
15	1:45	55	1.0	5.6	23.87	250.05
16	3:15	45	1.0	5.6	25.05	220.80
17	1:00	50	2.0	5.3	21.87	281.65
18	2:30	60	2.0	5.3	27.43	209.10
19	2:30	50	2.0	5.3	24.41	261.15
20	2:30	50	2.0	5.3	24.84	232.00
21	4:00	50	2.0	5.3	25.42	269.90
22	3:15	55	1.0	4.9	26.58	283.60
23	1:45	45	1.0	4.9	23.82	216.35
24	2:30	50	0.5	5.3	23.49	245.10
25	3:15	55	1.0	4.9	27.03	291.80
26	3:15	45	1.0	5.6	24.89	302.20
27	1:45	45	2.5	4.9	23.90	221.20
28	3:15	55	2.5	4.9	25.30	291.95
29	1:45	55	1.0	5.6	24.20	271.10

**Table 3:** The pH results during EAE extract fermentation with TKG.

	Time, h				
	0	24	48	72	98
pH	4.95 ± 0.020 c	4.68 ± 0.021 a	4.71 ± 0.041 a	4.81 ± 0.013 b	4.82 ± 0.042 b

Values represent mean ± standard deviation (*n* = 3). Different letters in the same column indicate significant differences (one-way ANOVA and Tukey’s HSD test, *p* < 0.05).

## Data Availability

All data generated during this study are included in this article.

## References

[1] Li T.S. (2004). 2. Taxinomie, répartition naturelle et botanique. Production et Utilisation de l’Argousier (Hippophae rhamnoides L.).

[2] Letchamo W., Ozturk M., Altay V., Musayev M., Mamedov N.A., Hakeem K.R. (2018). An alternative potential natural genetic resource: Sea buckthorn [*Elaeagnus rhamnoides* (syn.: *Hippophae rhamnoides*)]. Global Perspectives on Underutilized Crops.

[3] Bartish I.V. (2016). An ancient medicinal plant at the crossroads of modern horticulture and genetics: Genetic resources and biotechnology of sea buckthorn (*Hippophae* L., Elaeagnaceae). Gene Pool Divers. Crop Improv..

[4] Wang Z., Zhao F., Wei P., Chai X., Hou G., Meng Q. (2022). Phytochemistry, health benefits, and food applications of sea buckthorn (*Hippophae rhamnoides* L.): A comprehensive review. Front. Nutr..

[5] Żuchowski J. (2023). Phytochemistry and pharmacology of sea buckthorn (*Elaeagnus rhamnoides*; syn. *Hippophae rhamnoides*): Progress from 2010 to 2021. Phytochem. Rev..

[6] Gradt I., Kühn S., Mörsel J.-T., Zvaigzne G. (2017). Chemical composition of sea buckthorn leaves, branches and bark. Proc. Latv. Acad. Sci. Sect. B Nat. Exact. Appl. Sci..

[7] Sytařová I., Orsavová J., Snopek L., Mlček J., Byczyński Ł., Mišurcová L. (2020). Impact of phenolic compounds and vitamins C and E on antioxidant activity of sea buckthorn (*Hippophaë rhamnoides* L.) berries and leaves of diverse ripening times. Food Chem..

[8] Wang K., Xu Z., Liao X. (2022). Bioactive compounds, health benefits and functional food products of sea buckthorn: A review. Crit. Rev. Food Sci. Nutr..

[9] Raudone L., Puzerytė V., Vilkickyte G., Niekyte A., Lanauskas J., Viskelis J., Viskelis P. (2021). Sea buckthorn leaf powders: The impact of cultivar and drying mode on antioxidant, phytochemical, and chromatic profile of valuable resource. Molecules.

[10] He Q., Yang K., Wu X., Zhang C., He C., Xiao P. (2023). Phenolic compounds, antioxidant activity and sensory evaluation of sea buckthorn (*Hippophae rhamnoides* L.) leaf tea. Food Sci. Nutr..

[11] Li Y., Liu Q., Wang Y., Zu Y., Wang Z., He C., Xiao P. (2021). Application and modern research progress of sea buckthorn leaves. Zhongguo Zhong Yao Za Zhi.

[12] Saracila M., Untea A.E., Panaite T.D., Varzaru I., Oancea A., Turcu R.P., Vlaicu P.A. (2022). Effects of supplementing sea buckthorn leaves (*Hippophae rhamnoides* L.) and chromium (III) in broiler diet on the nutritional quality and lipid oxidative stability of meat. Antioxidants.

[13] Marić M., Grassino A.N., Zhu Z., Barba F.J., Brnčić M., Brnčić S.R. (2018). An overview of the traditional and innovative approaches for pectin extraction from plant food wastes and by-products: Ultrasound-, microwaves-, and enzyme-assisted extraction. Trends Food Sci. Technol..

[14] Hernández Becerra E., De Jesús Pérez López E., Zartha Sossa J.W. (2021). Recovery of biomolecules from agroindustry by solid-liquid enzyme-assisted extraction: A review. Food Anal. Methods.

[15] Costa J.R., Tonon R.V., Cabral L., Gottschalk L., Pastrana L., Pintado M.E. (2020). Valorization of agricultural lignocellulosic plant byproducts through enzymatic and enzyme-assisted extraction of high-value-added compounds: A Review. ACS Sustain. Chem. Eng..

[16] Patra A., Abdullah S., Pradhan R.C. (2022). Review on the extraction of bioactive compounds and characterization of fruit industry by-products. Bioresour. Bioprocess..

[17] Yuliarti O., Goh K.K., Matia-Merino L., Mawson J., Brennan C. (2015). Extraction and characterisation of pomace pectin from gold kiwifruit (*Actinidia chinensis*). Food Chem..

[18] Song Y., Han A., Park S., Cho C., Rhee Y., Hong H. (2020). Effect of enzyme-assisted extraction on the physicochemical properties and bioactive potential of lotus leaf polysaccharides. Int. J. Biol. Macromol..

[19] Štreimikytė P., Urbonavičienė D., Balčiūnaitienė A., Viškelis P., Viškelis J. (2021). Optimization of the multienzyme-assisted extraction procedure of bioactive compounds extracts from common buckwheat (*Fagopyrum esculentum* M.) and evaluation of obtained extracts. Plants.

[20] Streimikyte P., Viskelis P., Viskelis J. (2022). Enzymes-assisted extraction of plants for sustainable and functional applications. Int. J. Mol. Sci..

[21] Diez-Ozaeta I., Astiazaran O.J. (2022). Fermented foods: An update on evidence-based health benefits and future perspectives. Food Res. Int..

[22] Włodarczyk M., Śliżewska K. (2021). Obesity as the 21st Century’s major disease: The role of probiotics and prebiotics in prevention and treatment. Food Biosci..

[23] Pimentel T.C., de Assis B.B.T., dos Santos Rocha C., Marcolino V.A., Rosset M., Magnani M. (2022). Prebiotics in non-dairy products: Technological and physiological functionality, challenges, and perspectives. Food Biosci..

[24] Wastyk H.C., Fragiadakis G.K., Perelman D., Dahan D., Merrill B.D., Feiqiao B.Y., Topf M., Gonzalez C.G., Van Treuren W., Han S. (2021). Gut-microbiota-targeted diets modulate human immune status. Cell.

[25] Kotzekidou P. (2020). Improved traditional fermented foods of the Mediterranean Region–Health benefits as functional foods. Functional Foods and Biotechnology.

[26] Gao W., Zhang L. (2019). Comparative analysis of the microbial community composition between Tibetan kefir grains and milks. Food Res. Int..

[27] Liu Y., Zheng Y., Yang T., Mac Regenstein J., Zhou P. (2022). Functional properties and sensory characteristics of kombucha analogs prepared with alternative materials. Trends Food Sci. Technol..

[28] Streimikyte P., Kailiuviene J., Mazoniene E., Puzeryte V., Urbonaviciene D., Balciunaitiene A., Liapman T.D., Laureckas Z., Viskelis P., Viskelis J. (2022). The Biochemical alteration of enzymatically hydrolysed and spontaneously fermented oat flour and its impact on pathogenic bacteria. Foods.

[29] Zeng X., Wang Y., Jia H., Wang Z., Gao Z., Luo Y., Sheng Q., Yuan Y., Yue T. (2022). Metagenomic analysis of microflora structure and functional capacity in probiotic Tibetan kefir grains. Food Res. Int..

[30] Zheng Y., Lu Y., Wang J., Yang L., Pan C., Huang Y. (2013). Probiotic properties of Lactobacillus strains isolated from Tibetan kefir grains. PLoS ONE.

[31] Chen Z., Liu T., Ye T., Yang X., Xue Y., Shen Y., Zhang Q., Zheng X. (2021). Effect of lactic acid bacteria and yeasts on the structure and fermentation properties of Tibetan kefir grains. Int. Dairy J..

[32] Plessas S., Nouska C., Mantzourani I., Kourkoutas Y., Alexopoulos A., Bezirtzoglou E. (2016). Microbiological exploration of different types of kefir grains. Fermentation.

[33] Streimikyte P., Balciunaitiene A., Liapman T.D., Streimikyte-Mockeliune Z., Puzeryte V., Borkertas S., Viskelis P., Viskelis J. (2022). Enzymatically hydrolysed common buckwheat (*Fagopyrum esculentum* M.) as a fermentable source of oligosaccharides and sugars. Appl. Sci..

[34] Ricci A., Bertani G., Maoloni A., Bernini V., Levante A., Neviani E., Lazzi C. (2021). Antimicrobial activity of fermented vegetable byproduct extracts for food applications. Foods.

[35] Ricci A., Bernini V., Maoloni A., Cirlini M., Galaverna G., Neviani E., Lazzi C. (2019). Vegetable by-product lacto-fermentation as a new source of antimicrobial compounds. Microorganisms.

[36] Cruz-Casas D.E., Aguilar C.N., Ascacio-Valdés J.A., Rodríguez-Herrera R., Chávez-González M.L., Flores-Gallegos A.C. (2021). Enzymatic hydrolysis and microbial fermentation: The most favorable biotechnological methods for the release of bioactive peptides. Food Chem. Mol. Sci..

[37] Singleton V.L., Orthofer R., Lamuela-Raventós R.M. (1999). Analysis of total phenols and other oxidation substrates and antioxidants by means of Folin-Ciocalteu reagent. Meth. Enzymol..

[38] Bobinaitė R., Viškelis P., Venskutonis P.R. (2012). Variation of total phenolics, anthocyanins, ellagic acid and radical scavenging capacity in various raspberry (*Rubus* spp.) cultivars. Food Chem..

[39] Qadir R., Anwar F., Gilani M.A., Zahoor S., ur Rehman M.M., Mustaqeem M. (2019). RSM/ANN based optimized recovery of phenolics from mulberry leaves by enzyme-assisted extraction. Czech J. Food Sci..

[40] Syrpas M., Valanciene E., Augustiniene E., Malys N. (2021). Valorization of Bilberry (*Vaccinium myrtillus* L.) Pomace by enzyme-assisted extraction: Process optimization and comparison with conventional solid-liquid extraction. Antioxidants.

[41] Qadir R., Anwar F., Gilani M.A., Yaqoob M.N., Ahmad M. (2019). Enzyme-assisted extraction for optimized recovery of phenolic bioactives from *Peganum hermala* leaves using response surface methodology. Curr. Top. Nutraceutical Res..

[42] Bisswanger H. (2014). Enzyme assays. Perspect. Sci..

[43] Islam M.R., Kamal M.M., Kabir M.R., Hasan M.M., Haque A.R., Hasan S.K. (2023). Phenolic compounds and antioxidants activity of banana peel extracts: Testing and optimization of enzyme-assisted conditions. Meas. Food.

[44] Gligor O., Mocan A., Moldovan C., Locatelli M., Crișan G., Ferreira I.C. (2019). Enzyme-assisted extractions of polyphenols–A comprehensive review. Trends Food Sci. Technol..

[45] Fernandes A., Mateus N., de Freitas V. (2023). Polyphenol-dietary fiber conjugates from fruits and vegetables: Nature and biological fate in a food and nutrition perspective. Foods.

[46] Wang Z., Li S., Ge S., Lin S. (2020). Review of distribution, extraction methods, and health benefits of bound phenolics in food plants. J. Agric. Food Chem..

[47] Thite V.S., Nerurkar A.S. (2019). Valorization of sugarcane bagasse by chemical pretreatment and enzyme mediated deconstruction. Sci. Rep..

[48] Dong M., Wang S., Xu F., Wang J., Yang N., Li Q., Chen J., Li W. (2019). Pretreatment of sweet sorghum straw and its enzymatic digestion: Insight into the structural changes and visualization of hydrolysis process. Biotechnol. Biofuels.

[49] Rafińska K., Wrona O., Krakowska-Sieprawska A., Walczak-Skierska J., Kiełbasa A., Rafiński Z., Pomastowski P., Kolankowski M., Buszewski B. (2022). Enzyme-assisted extraction of plant material–New functional aspects of the process on an example of *Medicago sativa* L.. Ind. Crops Prod..

[50] Łopusiewicz Ł., Śmietana N., Paradowska D., Drozłowska E. (2022). Black cumin (*Nigella sativa* L.) seed press cake as a novel material for the development of new non-dairy beverage fermented with kefir grains. Microorganisms.

[51] Rodrigues D., Walton G., Sousa S., Rocha-Santos T.A., Duarte A.C., Freitas A.C., Gomes A.M. (2016). In vitro fermentation and prebiotic potential of selected extracts from seaweeds and mushrooms. LWT.

[52] Gómez-García R., Vilas-Boas A.A., Oliveira A., Amorim M., Teixeira J.A., Pastrana L., Pintado M.M., Campos D.A. (2022). Impact of simulated human gastrointestinal digestion on the bioactive fraction of upcycled pineapple by-products. Foods.

[53] Harper A.R., Dobson R.C., Morris V.K., Moggré G. (2022). Fermentation of plant-based dairy alternatives by lactic acid bacteria. Microb. Biotechnol..

[54] Montemurro M., Pontonio E., Coda R., Rizzello C.G. (2021). Plant-based alternatives to yogurt: State-of-the-art and perspectives of new biotechnological challenges. Foods.

[55] EFSA Panel on Dietetic Products, Nutrition and Allergies (NDA) (2010). Scientific Opinion on the substantiation of health claims related to live yoghurt cultures and improved lactose digestion (ID 1143, 2976) pursuant to Article 13 (1) of Regulation (EC) No 1924/2006. EFSA J..

[56] Jan G., Tarnaud F., do Carmo F.L.R., Illikoud N., Canon F., Jardin J., Briard-Bion V., Guyomarc’h F., Gagnaire V. (2022). The stressing life of *Lactobacillus delbrueckii* subsp. *bulgaricus* in soy milk. Food Microbiol.

[57] Rezac S., Kok C.R., Heermann M., Hutkins R. (2018). Fermented foods as a dietary source of live organisms. Front. Microbiol..

[58] Dou Z., Chen C., Fu X. (2019). Bioaccessibility, antioxidant activity and modulation effect on gut microbiota of bioactive compounds from *Moringa oleifera* Lam. leaves during digestion and fermentation in vitro. Food Funct..

[59] Kaewkod T., Bovonsombut S., Tragoolpua Y. (2019). Efficacy of kombucha obtained from green, oolong, and black teas on inhibition of pathogenic bacteria, antioxidation, and toxicity on colorectal cancer cell line. Microorganisms.

[60] Alimentarius C. (2015). Codex Standard for Fermented Milks (CODEX STAN 243-2003). Milk and Milk Products.

[61] Łopusiewicz Ł., Drozłowska E., Siedlecka P., Mężyńska M., Bartkowiak A., Sienkiewicz M., Zielińska-Bliźniewska H., Kwiatkowski P. (2019). Development, characterization, and bioactivity of non-dairy kefir-like fermented beverage based on flaxseed oil cake. Foods.

[62] Spizzirri U.G., Loizzo M.R., Aiello F., Prencipe S.A., Restuccia D. (2023). Non-dairy kefir beverages: Formulation, composition, and main features. J. Food Compos. Anal..

[63] Sousa S., Pinto J., Pereira C., Malcata F.X., Pacheco M.B., Gomes A.M., Pintado M. (2015). In vitro evaluation of yacon (*Smallanthus sonchifolius*) tuber flour prebiotic potential. Food Bioprod. Process.

